# Game of Tissues: How the Epidermis Thrones *C. elegans* Shape

**DOI:** 10.3390/jdb8010007

**Published:** 2020-03-09

**Authors:** Cátia A. Carvalho, Limor Broday

**Affiliations:** Department of Cell and Developmental Biology, School of Medicine, Tel Aviv University, Tel Aviv 69978, Israel; carvalhoc@mail.tau.ac.il

**Keywords:** epithelial morphogenesis, epidermal-neuroblast axis, epidermal-muscle axis, *C. elegans*

## Abstract

The versatility of epithelial cell structure is universally exploited by organisms in multiple contexts. Epithelial cells can establish diverse polarized axes within their tridimensional structure which enables them to flexibly communicate with their neighbors in a 360° range. Hence, these cells are central to multicellularity, and participate in diverse biological processes such as organismal development, growth or immune response and their misfunction ultimately impacts disease. During the development of an organism, the first task epidermal cells must complete is the formation of a continuous sheet, which initiates its own morphogenic process. In this review, we will focus on the *C. elegans* embryonic epithelial morphogenesis. We will describe how its formation, maturation, and spatial arrangements set the final shape of the nematode *C. elegans*. Special importance will be given to the tissue-tissue interactions, regulatory tissue-tissue feedback mechanisms and the players orchestrating the process.

## 1. The Birth of the Stars

The *C. elegans* epidermal tissue derives from the ectodermal germ-layer and emerges around 240 min after first cell division. The epithelial cells are born at the dorsal side of the embryo, with the first-born cells composing most of the epidermal sheet, and the second round of epithelial births giving rise to the head and tail regions [1,2]. Ensuing development will establish different transcriptional programs within the epidermal tissue, patterning it into three main sub-tissues: (1) The dorsal cells, which are initially located in two adjacent rows in the dorsal midline, (2) lateral/seam cells which line the dorsal cells and separate them from (3) the ventral cells located at the outermost edge of the epithelial sheet that later will enclose the embryo ventrally [1,2]. To accomplish the patterning into epidermal sub-tissues, a cascade of transcriptional activation and negative feedback is triggered, and while much of the pathway is characterized, several players have not yet been identified [3,4,5,6,7,8]. At the top of the hierarchy sits ELT-1, a GATA transcription factor that is necessary and sufficient to specify most epidermal cell features [8,9]. The absence of ELT-1 results in embryonic lethality due to impaired morphogenesis, as epidermal cells are not generated [6]. ELT-1 subsequently activates the expression of two other transcription factors that fine-tune the differentiation of the epithelial cells into subtypes, among them and of particular importance are LIN-26 and ELT-3 [6,7,10,11].

## 2. Morphogenesis of the Epidermal Tissue

LIN-26 induces the expression of the junctional proteins DLG-1 and AJM-1 (DAC complex), which initiate epithelial differentiation [7,12]. The DAC complex localizes at a dense spot along with the classic cadherin complex (CCC complex) composed by HMR-1 (E-CAD), HMP-1 (α-CAT), and HMP-2 (β-CAT) and together form the apical *C. elegans* junction (CeAJ) [13,14,15]. The CeAJ performs both the paracellular barrier and the adhesion functions which are found in distinct structures in epithelial cells across evolution [15,16,17]. The establishment of the CeAJs initiates the emergence of the epithelial cell shape and their functional segregation. The epithelial shape evolves during development, adapting to incorporate signals and forces from internal tissues, ultimately shaping the form of the worm. This process is collectively called morphogenesis and can be divided into three main events: dorsal intercalation, ventral enclosure, and elongation [1]. Each will be described below.

### 2.1. Dorsal Intercalation

**Figure 1:**
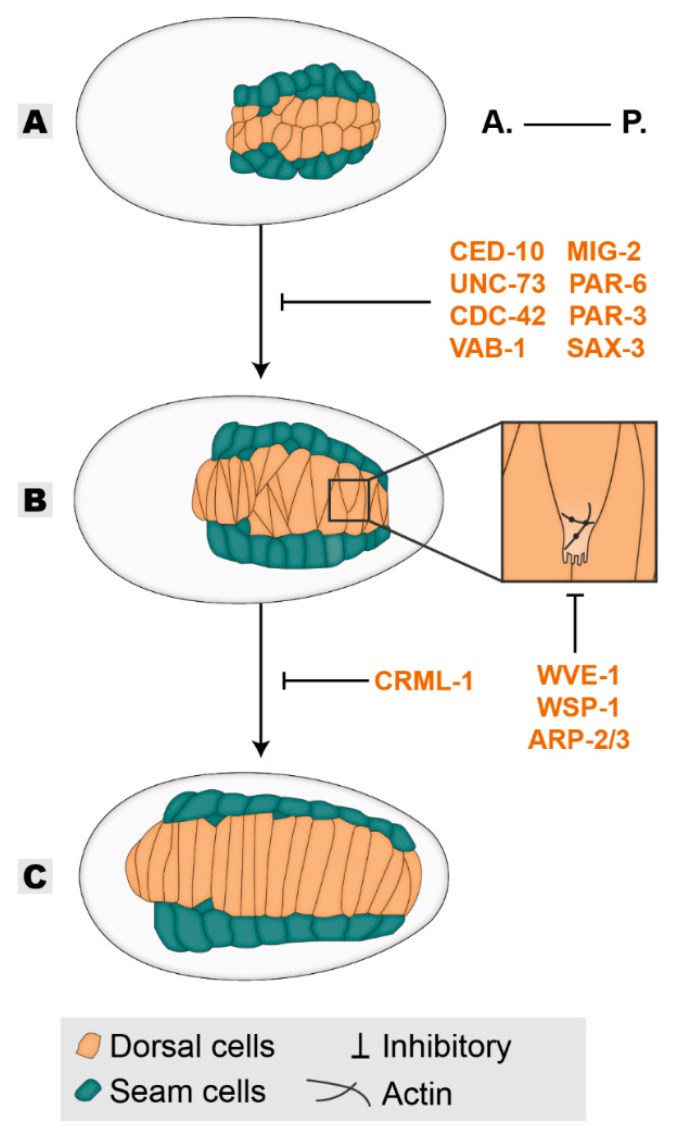
Dorsal intercalation. Epithelial cells are born dorsally as two rows (**A**), the dorsal cells reorganize their shape to intercalate within themselves (**B**) covering the dorsal length of the embryo (**C**). Players in orange are required in the epidermis for dorsal intercalation. All inhibitory signs are representative of the outcome of the absence of a player. Embryos oriented with anterior (A.) on the left and posterior (P.) on the right.

After their birth at the dorsal side of the embryo, dorsal epidermal subtype cells accommodate rearrangements in their organization. Initially, they appear organized into two rows of ten cells located at the posterior part of the embryo (Figure 1A). Within approximately 90 min, the rounded cells will become wedged towards the dorsal midline and in the direction of their migration, initiating intercalation with the opposing dorsal row (Figure 1B). Elongation of the wedged tips will continue until contact is made with the contralateral seam cell row, where new junctions are established, forming a single row of dorsal cells [1,18,19]. Ultimately, this process results in the spanning of dorsal cells over the entire dorsal length of the embryo akin to convergent-extension mechanisms in other organisms (Figure 1C) [20]. This intercalation is highly reliant on cell-autonomous events, as it is not dependent on the existence of lateral seam cells, muscle cells, or the sealing properties of the CeAJs [15,21,22]. It does, however, rely on an adaptively responding cytoskeleton for its successful completion [21]. During intercalation, dorsal cells generate basolateral protrusive extensions on their medial side that guide them past the likewise protrusive edges of axial opposing neighbors. Recent technical advances allowed the dissection of the players involved in the control of the cytoskeleton-based protrusion formation [19]. CRML-1 (CARMIL), which acts as an inhibitor of actin polymerization, in this context drives the polarized action of the UNC-73 GEF (TRIO) by initially inhibiting its activity at the lateral and rear end of the intercalating cell. At the leading edge, uninhibited UNC-73 activates the GTPases CED-10 (RAC-1) and MIG-2 (RHOG) that act through WVE-1 (WAVE) and WSP-1 (WASP) respectively, to promote protrusion formation through branched-actin polymerization mediated by the ARP-2/3 nucleation complex (Figure 1B inset) [18,19,23,24]. Once the extended tip of the dorsal cell reaches the contralateral seam cell, CRML-1 localizes at the leading edge and inhibits further UNC-73-mediated protrusive activity. Therefore, CRML-1 promotes polarized protrusion formation during the elongation of the intercalating cells [19]. In addition to the formation of planar polarized protrusions, proper intercalation also requires the polarized orientation of protruding tips [25]. The CDC-42 GTPase promotes protrusion formation and also dictates its orientation. During tip formation, active CDC-42 localizes at the leading edge of the migrating dorsal cell in a PAR-6 and VAB-1-dependent manner to steer the protruding tips of same-side migrating cells away from one another. This property enables interdigitation and inhibits co-migration [25]. The success of this morphogenetic process is of crucial importance to subsequent events, as its failure negatively impacts the formation of uniformly distributed actin circumferential across the dorsal epidermis and presumably affects epidermal elongation [22].

### 2.2. Ventral Enclosure

**Figure 2:**
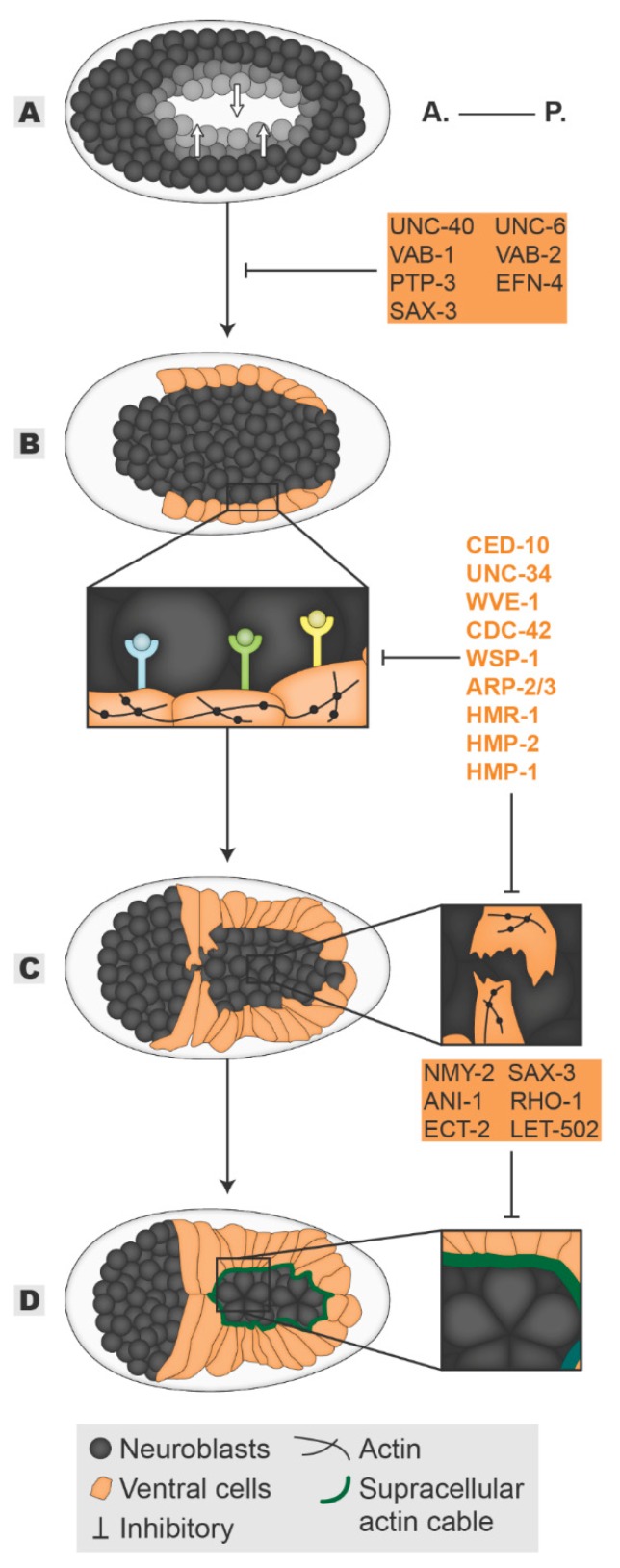
Ventral enclosure. Ventral enclosure encompasses the following “steps”: In the end of gastrulation, the neuroblasts close the ventral cleft (**A**) allowing the migration of the ventral epidermal cells (**B**). The “leading cells” initiate the migration (**C**) followed by the “pocket cells” (**D**), enclosing the embryo in a continuous epidermal layer. In each ventral enclosure “step”, players in grey and highlighted in orange are required in neuroblasts and epidermis, whereas players in orange are required in the epidermal tissue. All inhibitory signs are indicative of the outcome of the absence of a player. Embryos oriented with anterior (A.) on the left and posterior (P.) on the right.

Mid-way through dorsal intercalation, the ventral cells start to migrate. Within 40 min, the ventral cells extend over the neuroblasts, meeting at the ventral midline and enclosing the embryo in a process known as epiboly (Figure 2B–D) [21,26,27]. To achieve enclosure, ventral cells organize distinct cytoskeleton-driven processes to propel their migration: (1) The “leading cells” start ventral migration on the most anterior side of the embryo by organizing actin-rich broad lamellipodia extensions (Figure 2C inset); (2) the ventral posterior “pocket cells” close a ventral pocket by driving the shrinkage of a supracellular actin cable built at their most ventral edges through a mechanism resembling a “purse-string” (Figure 2D inset) [26,27]. After contralateral cell-cell contact is made, ventral cells cease migration and assemble new junctions, thereby establishing a continuous epidermal sheet framing the embryo. This process relies upon both cell-autonomous and non-autonomous mechanisms, and the latter will be described below. Seminal laser ablation experiments defined the initial migration led by the “leading cells” as well as the uninterruptedness of the “pocket cells” actin cable as the initial essential steps to ensure epidermal enclosure [27,28]. As expected, the master regulators of actomyosin dynamics CED-10, RHO-1(RHOA), and CDC42 GTPases have been shown to mediate both steps. In the “leading cells”, the usual suspects orchestrate lamellipodia protrusion formation: (1) the potential CED-10-WVE-1 axis is enhanced by WVE-1-dependent recruitment of UNC-34 (VASP) and (2) the CDC-42-WSP-1 axis magnifies the nucleation potential of the ARP-2/3 complex to form branched-actin filaments. Loss of any of these mediators results in an overall reduction of protrusive activity of the “leading cells” which either halt or slow down ventral migration causing ventral enclosure defects (Figure 2C inset) [24,29,30,31,32,33,34]. On the other hand, in the “pocket cells”, the axis ECT-2(RHOA GEF)-RHO-1-LET-502(ROCK) restricts protrusive ability while enhancing the actin motor non-muscle myosin II (NMY-2) contractility to ensure constriction of the actin ring, enclosing the ventral pocket (Figure 2D inset) [31,33,34,35]. Once the epidermal cells meet, new CeAJs must form. In agreement, the classical cadherin complex is essential for the establishment and maturation of the newly formed junctions as their absence leads to extruded embryonic content and an open epidermal sheet [15,16]. HMR-1 localization at the CeAJs is stabilized by SUMO-mediated regulation, while its connection to HMP-2 is regulated by phosphorylation [36,37]. In turn, HMP-1 bridges the junctions and the cytoskeleton by binding both HMP-2 and F-actin, strengthening the junction and enabling the epidermal sheet to resist increased embryonic tension [15,16,38,39,40].

#### Epidermal-Neuroblasts Axis

The successful ventral enclosure of the embryo is largely dependent on the substrate on which the epidermal cells migrate—the neuroblasts. During gastrulation, cells that will give rise to mesoderm and endoderm tissues ingress into the embryo, leaving a cellular void at the ventral side called the ventral cleft [41]. The neuroblasts skirting this cleft migrate towards the ventral midline, closing the gap and forming a continuous substrate. Once the ventral cleft is enclosed, the neuroblasts rearrange by forming two temporally sequential rosettes in an anterior-posterior direction. The rosettes are resolved through a convergent-extension mechanism enabling the formation of a single neuroblast row along the ventral midline (Figure 2D) [42,43]. Multiple studies have begun to uncover the interlinked relationship between the morphogenesis of neuronal and epidermal tissues. The initial neuroblast migration is dependent on classical axon guidance pathways. The Ephrin signaling is known to mediate short-range cell-cell bidirectional communication involved in neuronal guiding and sorting of mixed cell populations [44]. In accordance, loss of function of the *C. elegans* Ephrin receptor VAB-1 stalls neuroblasts in their migration path, resulting in a persistent open ventral cleft [45,46]. This provides the first evidence that epidermal enclosure depends on neuroblasts: despite both the Ephrin receptor and its ligand VAB-2 being expressed in the neuroblasts, their loss of function induces a non-autonomous effect on the ventral leading cells, halting their migration [46,47]. Two models were initially proposed to explain neuroblast-dependent epidermal enclosure—“steric hindrance” and “reverse signaling” (Figure 2A,B inset). In the “steric hindrance” model, the absence of a continuous or correctly oriented neuronal substrate over which epidermal cells migrate is considered the cause of the non-autonomous effect whereas in the “reverse signaling” model, it is the absence of directive cues provided by the neuroblasts that lie at the root of the migratory defect. As is more the rule than the exception, both models have been validated and are intertwined [42,46,48,49,50]. Multiple signaling pathways involved in neuronal migration guidance have corroborated a non-autonomous effect of neuroblasts on epidermal morphogenesis, supporting the “reverse signaling” model. Among them, functional perturbation of the Ephrin ligand EFN-4, the tyrosine phosphatase receptor PTP-3, the UNC-40/UNC-6 (Nectrin), and the SAX-3/SLT-1(ROBO/Slit) signaling pathways all resulted in persistent ventral cleft opening and halted ventral epidermal migration. In addition, their defects synergize with the phenotype observed for vab-1 [48,49,51,52]. Among these, SAX-3 is an exception: It acts both non-autonomously and autonomously as it is expressed also in the epidermal tissue. Loss of function of SAX-3 displays dorsal intercalation defects independent of its neuroblast expression, but interestingly, its notched head phenotype resulting from abnormal epithelial morphogenesis cannot be rescued by re-expression of SAX-3 in the epidermal cells [49]. One possibility is that SAX-3-mediated intercalation of neuroblasts during rosette resolution provides the appropriate neuronal substrate for oriented epidermal migration [42]. Despite mediating neuronal migration in parallel, the non-autonomous effects of these signaling pathways converge in the regulation of actin dynamics at the level of the epidermis (Figure 2B inset). Loss of function of SAX-3, VAB-1, and UNC-40 compromised the localization and levels of the actin regulators CED-10 and WVE-1 at the ventral edges of the “leading cells”. This results in an overall loss of ventrally polarized F-actin and diminished protrusive activity, accounting for their migratory defects [48]. The role of these signaling molecules on epidermal enclosure supports not only the “reverse signaling” model but also the “steric hindrance” model as an unclosed ventral cleft is often observed. The “steric hindrance” model was further supported when studies showed that the morphogenesis of the neuroblasts mechanically affects the enclosure of the epidermis. The initial supporting claim comes from Ikegami and colleagues who identified the formation of a neuroblast bridge spreading over the ventral pocket, which directs the movements of the overlying “pocket cells” towards the ventral midline. When this bridge does not form correctly, the “pocket cells” either fail to migrate or migrate too slowly, leaving gaps through which embryonic content can be extruded during subsequent development [50]. Furthermore, NMY-II was found to localize in a star-like pattern in the posterior neuroblasts where it participates in the resolution of the rosettes, which dictates the topology of the neuroblasts (Figure 2D inset). In agreement, loss of myosin activity in the neuroblasts leads to a failure in rosette formation and decreased neuroblast surface constriction, resulting in impaired or slowed ventral pocket closure [34,42]. Moreover, the ECT-2-RHO-1-LET-502 pathway, known to regulate myosin activity, also participates in positioning neuroblasts during migration. In accordance, loss of their function leads to a decreased accumulation of myosin foci at the neuroblasts resulting in an open ventral pocket, supporting the role of myosin activity in neuroblasts to drive epidermal enclosure [33,34]. Lastly, ANI-1 (anillin), a scaffold protein that bridges cytoskeleton components, also has a non-autonomous role in the epidermal enclosure. Its loss changes neuroblast surface topology as it generates multinucleated, misshapen and mispositioned neuroblasts. Additionally, the rate at which neuroblasts constrict their surface area is delayed. Consequently, epidermal cell migration is either halted or occurs slowly [33,34]. These, all together, support the notion that neuroblasts provide a mechanical support for epidermal morphogenesis [34].

### 2.3. Elongation

**Figure 3:**
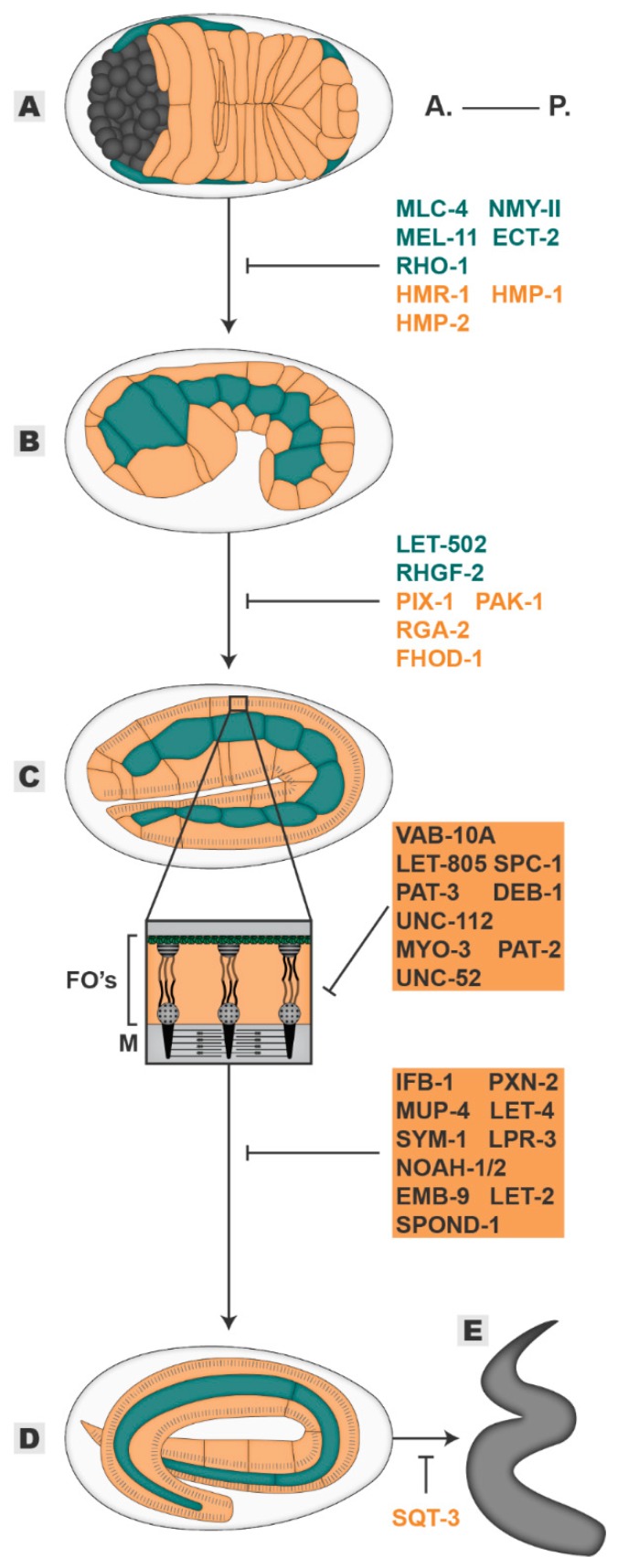
Elongation. The embryo progressively elongates from an initial 50 µm oval shape (**A**), through 1,5-fold (**B**), two-fold (**C**) and three-fold stages (**D**) until it hatches as a larva (**E**). Players in blue are required mainly in the seam cells, players in orange are required mainly in the dorsal/ventral cells, players in grey highlighted in orange are required in muscle and epidermal tissues. All inhibitory signs are representative of the outcome of the absence of a player. Embryos oriented with anterior (A.) on the left and posterior (P.) on the right. FOs—Fibrous Organelles, M—muscle.

As soon as the epidermis encloses, the embryo starts to elongate. This process is dependent on both the cell-autonomous and non-autonomous force generation and conduction that drives the squeezing of the embryonic contents from a 50 µm oval egg into a 200 µm cylindrical larvae (Figure 3). The elongation process can be divided into two main phases: (1) From one- to two-fold where elongation relies mostly on epidermal cell-autonomous actomyosin contractile forces, (2) and beyond the two-fold stage where it depends mainly upon cell non-autonomous forces generated by the underlying muscle tissue. In the first stage of elongation (one- to two-fold), the epidermal tissue undergoes a dramatic re-organization of its three-dimensional structure, deepening the heterogeneity among the epidermal subtypes. In the ventral cells, right after epidermal enclosure, the apposed membranes of the newly formed CeAJs fuse, initiating the generation of the epidermal syncytium. Subsequently, a wave of fusion events at the dorsal cells occurs in an anterior-posterior direction terminating by the two-fold stage2. The EFF-1 fusogen is both necessary and sufficient to drive membrane fusion [53]. Though not essential, epidermal cell fusions set the stage for future force-driven elongation as it renders the syncytium more plastic than its unfused version [53,54,55,56]. Concomitantly with fusion events in both dorsal and ventral cells, the assembly of the Fibrous Organelle (FO), a transversal junction running along the epidermal apical-basal axis, begins. Punctate structures are first observed around 400 min after first cleavage and progressively mature into anterior-posterior perpendicular stripes of FOs at the epidermal-muscle interface. These structures are composed of an apical and an apposed basal hemidesmosome-like junction (CeHD) connected by bundles of intermediate filaments [1,57]. On its apical side, the hemidesmosome connects the epidermis to the apical ECM/cuticle through MUP-4 and MUA-3 receptors while on the basal side myotactin connects the CeHD to the shared basal ECM between muscles and epidermis [58,59,60,61]. The connection of both CeHDs into a functional FO is mediated by the *C. elegans* spectraplakin VAB-10A that controls the attachment of both ends of intermediate filament bundles to both CeHDs (Figure 3C inset) [62]. Hence FOs physically link the epidermis to the underlying muscles permitting the transmission of the force generated in the musculature throughout the epidermis which is central in the second stage of elongation [13,57,61]. On the other hand, the seam cell branch of the epithelium does not fuse during elongation. In these cells, the transcriptional program controlled by the CEH-16-ELT-5/6 axis represses the expression of both ELT-3 and EFF-1, both of which are required later for cuticle secretion and fusion, respectively [4,5]. As a result, the seam cells remain individually connected by CeAJs and form two rows of cells along the entire length of the embryo, segregating dorsal and ventral compartments. This individuality allows them to establish a polarized axis at the plane of the epithelium. The localization of the PAR module -PAR-3, PAR-6, and PKC (aPKC)- is redistributed throughout the first phase of elongation, shifting from a uniform localization along all CeAJs to a restricted localization at CeAJs shared only by seam cells, establishing planar polarization [63]. This will become important in the second stage of elongation. The epidermal surfaces lining the membrane borders also undergo specialization as they produce apical and basal extracellular matrixes (ECM). The epidermal basal ECM, or basal lamina, is secreted and shared by both epidermis and muscle tissues. In terms of composition, the basal lamina is similar to basal ECMs found in other organisms, composed of collagen type IV (EMB-9 and LET-2), peroxidases (PXN-2), proteoglycans (UNC-52) or the F-Spondin (Spond-1) family [64,65,66,67,68,69]. In turn, the apical ECM or embryonic sheath is only secreted by the epidermis and locates in close apposition to the underlying apical actin cytoskeleton. Likewise, it has a similar composition to other apical ECMs with leucine-rich proteins (SYM-1, LET-4, and EGG-6), zona-pellucida containing proteins (FBN-1, NOAH-1, NOAH-2) and lipocalins (LPR-1 and LPR-3) [70,71,72,73]. Both matrixes line exposed epidermal surfaces and are essential to withstand, mold and hold epidermal cell shapes as they go through dynamic changes throughout elongation [70,71,72,74,75].

#### 2.3.1. 1st Stage of Elongation

In the first stage of elongation, the extension of the embryo from 50 to 100 µm in length is achieved through the simultaneous constriction and lengthening of perpendicular epidermal cell axes (Figure 3A–C) [1,26]. Although the organization of the epidermal cytoskeleton differs between the dorsal-ventral and the seam cell compartments, pharmacological and genetic analyses propose that both differential cytoskeleton organizations are the essential elements that drive the first stage of elongation [75,76,77,78]. In dorsal-ventral cells, by the time the embryo reaches 1,3-fold in length, actin cables start to assemble along the dorsal-ventral axis. Until the two-fold stage, they progressively mature into thick arrays that run from dorsal/ventral-seam CeAJs through CeHDs to the opposite dorsal/ventral-seam CeAJs [75]. Concurrently, in the seam cells, actin filaments begin to gradually display a dorsal-ventral orientation until the two-fold stage, albeit less organized in comparison to dorsal-ventral cells [54]. On the other hand, the actin myosin motor NMY-II despite lacking a biased orientation within cells, displays polarized activity within the epidermal subtypes: (1) it is highly active in the seam cells, as synergy between RHGF-2(RHO1 GEF)-RHO-1-LET-502 and CED-10-PIX-1(CDC42/RAC GEF)-PAK-1(p-21 activated kinase) pathways antagonize MEL-11 (myosin phosphatase) activity but phosphorylate and activate the non-muscle myosin regulatory subunit (MLC-4); (2) its activity in dorsal-ventral cells is kept low through the action of RGA-2(RHO GAP) which inhibits RHO-1-LET-502 axis allowing MEL-11 activation (Figure 3A,B) [76,77,78,79,80,81,82,83]. How is this asymmetric distribution of actomyosin organization and activity translated into elongation? In the seam cells, high myosin contractile activity induces cellular compressive forces at the apical medial plane, generating increasing mechanical stress. This mechanical stress is progressively distributed in a dorsal-ventral orientation accompanying the ongoing alignment of the actin filaments in the same axis. Consequently, force anisotropy is generated leading to the contraction of the seam cells along the dorsal-ventral axis. Due to volume conservation, elongation of the seam cells along their anterior-posterior axis takes place. At the same time, the seam cell CeAJs propagate the increasing mechanical stress into the dorsal-ventral compartments, subjecting it to tensile forces. Moreover, hydrostatic pressure resulting from compression of internal tissues also increases. Therefore, despite possessing low myosin contractile activity, the dorsal-ventral subtypes integrate the received forces to drive the shortening of their circumference. Given that the increasing thickness of the dorsal-ventral oriented actin cables throughout development induces increased stiffness, dorsal-ventral cells resist force deformation in their dorsal-ventral axis and elongate in the anterior-posterior axis. Hence, the heterogenous epidermal subtypes coordinate their cytoskeleton responses to guide oriented deformation [54]. However, force-driven cell shape changes must be balanced with proper conduction and relief of these forces to avoid concentrated tension spots that might otherwise rupture the tissue. For example, when myosin contractile activity is decreased, embryos do not have the strength to elongate and so arrest in early elongation. However, if myosin contractility is hyperactivated, embryos rupture due to unrelieved tension [76,77,78,79,80]. The intimate connection between the cytoskeleton and junctions is central in achieving this balance. In HMP-1 mutants, the connection between the CeAJs and the actin cytoskeleton is abrogated, resulting in reduced accumulation of cortical junctional actin and fractured attachment of the circumferential actin cables to the CeAJs. Consequently, cell-cell adhesion is unbalanced by an unequal distribution of pulling forces. This lowers resistance to tension, resulting in rupture and aborted elongation [15,16,84]. In the same line, Martin and colleagues propose that differential employment of actomyosin regulatory pathways by the epidermal subtypes may simultaneously relieve tension. They showed that dorsal cells use the RAC-1 mediated pathway to generate lamellipodia-like protrusions below the level of the CeAJs towards the seam cells while the seam cells use the RHO-1 mediated pathway to induce the formation of amoeboid-like protrusions in the direction of the dorsal cells. At the same time, the myosin activity controlled by these pathways regulates the remodeling of the CeAJs, where an increased myosin activity correlates with a decreased junctional length [84,85]. Thus, while the cells are restructuring their apical junctions, the basolateral membranes are relieving the tension generated in CeAJs through protrusive activity [85]. In accordance, a FRET-based force sensor placed in the HMP-1 gene, revealed that the tension exerted at the CeAJs decrease by 1.3- to 1.5-fold even though cells are experiencing high actomyosin contraction, supporting the idea that membrane protrusion acts as a tension relieving mechanism [54,84,85,86]. Adding to tension-relieving mechanisms, junction strengthening also provides a means by which cells can reinforce tension resistance. The microtubule cytoskeleton has been reported to mediate junctional strengthening. Although microtubules have a minor role in supporting elongation, they are involved in the transport of HMR-1 to the junctions, promoting a higher turnover and availability of HMR-1 at the CeAJs and securing their integrity and remodeling [87,88].

#### 2.3.2. 2nd Stage of Elongation

When the now tubular embryo reaches twice the size of the eggshell, actomyosin-driven forces generated by the seam cells are no longer enough to propel further squeezing. At this stage, the epidermal sheet assumes a more passive role in force generation, being mainly required to properly relay the forces exerted upon it. The musculature underlying the epidermis becomes the main force generator as its contractile capacity initiates around the two-fold stage (Figure 3C inset).

##### Epidermal-Muscle Axis

Body wall muscle cells are born adjacent to lateral seam cells around 290 min after fertilization [89,90,91]. During epidermal dorsal intercalation and ventral enclosure, the muscle cells migrate from their lateral positions towards both the dorsal and ventral sides of the embryo. Here they form four quadrants of muscles, two in each dorsal and ventral epidermal compartment, spanning the entire length of the embryo. When muscle cells assume their final positions, physical contacts between muscle cells and the overlying epidermis are initiated (FOs formation). At the level of the muscle membrane, adjacent to the basal lamina, the muscle cells form organized contractile units composed of myosin and actin—the sarcomeres—which power the contraction of the muscles (Figure 3C inset). The contractile capacity of the muscles reaches its maximum potential by the two-fold stage [89,92]. Muscle contractions assume the primary role in driving elongation from the two-fold stage onwards, with the epidermis assuming a more passive role through the channeling of muscle-generated force. The first observations supporting this idea came from mutagenesis screens aimed at identifying components required for the formation and maintenance of the body wall muscle cells [93]. The inability of the embryo to initiate contraction at the two-fold stage—called the paralyzed arrest at two-fold (PAT) phenotype—identified genes required for muscle formation such as the integrin heterodimer (PAT-3 and PAT-2), myosin (MYO-3), kindlin (UNC-112), or vinculin (DEB-1) [90,93,94,95,96,97]. Strikingly, these mutants also exhibited stalled embryonic elongation, frequently hatching with the size of a two-fold embryo, suggestive of a role for muscle function in driving the elongation of the embryo [93]. Efforts have been made to understand how muscle cells drive elongation beyond the two-fold stage, both mechanically and biochemically. The current model proposes that muscle-mediated epidermal elongation occurs through the successive stabilization of progressively anterior-posterior elongated intermediate epidermal shapes akin to a ratchet-like mechanism [98]. Muscle contractions occur nonsynchronously every few seconds (1–5 s) [98,99]. These contractions compress the epidermis locally through their attachment to the CeHDs and induce bends in the dorsal-ventral circumferential actin cables. The bend of the actin cables is strong enough to stimulate the action of actin severing proteins, which presumably leads to their shortening. During the relaxation state, the concerted action of the FHOD-1 (formin) bundling activity and the cytoskeleton scaffold SPC-1 (α-spectrin) stabilizes the shortened actin cables, reducing the cell length along their dorsal-ventral axis. Repeated muscle contraction cycles progressively induce shorter actin cables, which drives the reduction of the embryo circumference along the dorsal-ventral axis while elongating along the anterior-posterior direction [98,100,101]. To enable this muscle-driven epidermal elongation a four-piece functional module must assemble: 1: A contractile muscle tissue, 2: An adhesive substrate composed of basal ECM and FOs, 3: An integral epidermal tissue and 4: A relaying apical ECM. On one hand, integrin-mediated attachments connect the sarcomeres to the shared muscle-epidermis basal lamina [97]. On the other hand, the myotactin (LET-805) receptor connects the epidermal FOs and cytoskeleton to the same basal lamina [58]. This enables a continuous circuit through which force generated in the muscles is transmitted to the epidermal cytoskeleton. Interestingly, the maintenance of this continuous circuit is autonomously dependent on both muscle and epidermal cells and these tissues feedback bi-directionally and positively on one another. The muscle-epidermal directional feedback primarily involves the remodeling and stabilization of the FOs through mechanotransduction mechanisms. Laser ablation experiments demonstrated that intact muscle cells attached to the epidermis are required for the localization and stabilization of the myotactin basal receptor at the CeHDs [58]. In addition, their functional sarcomeres also strengthen the connection between apical and basal CeHDs. The contraction of the muscles induces myotactin and VAB-10A-dependent recruitment and maintenance of the G-protein receptor GIT-1 at the CeHDs [99,102]. Here, GIT-1 promotes the activation of the PIX-1-CED-10-PAK-1 axis, resulting in the phosphorylation of the intermediate filament component IFA-3 and its stable localization between both CeHDs. In addition, the IFB-1 component of the intermediate filaments is correctly polymerized at the FOs through SUMO-mediated regulation [103]. Ultimately this secures and reinforces the force transmission path between muscles and epidermis [99]. A second way muscles establish their force transmission path is through the regulation of the constituents of the basal lamina, which, by being sandwiched between both tissues, grant adhesive properties. The major structural component of the basal ECM is the proteoglycan UNC-52, presumably secreted by both muscle and epidermal cells and bi-directionally promotes their physical connection. Embryos with mutations in UNC-52 arrest development at two-fold stage and are paralyzed. Laser ablation of muscle cells results in a disrupted UNC-52 localization and as a consequence muscles no longer polarize or generate force [93,104]. Conversely, the alternative slicing of UNC-52 into its isoforms occurs at the level of the epidermis through MEC-8 and CCAR-1, which, when defective, disrupts CeHDs formation and maintenance [65,105]. Additional components of the basal ECM must maintain the muscle-epidermal contacts further into development (Figure 3D). The muscle secreted proteins, collagen type IV (EMB-9 and LET-2) and SPOND-1, and the epidermal secreted PXN-2 peroxidase are required for proper elongation beyond the 2,5-fold stage [64,66,67,93]. Characteristically, loss of function of these proteins show a progressive paralysis of the embryo and halted elongation by the three-fold stage despite normal initiation of muscle contractions. Moreover, it is accompanied by a detachment of the muscles from the epidermis, consistent with their role in maintaining the physical connection between both tissues. Interestingly, despite being essential for the elongation process, the gain in the function of cell matrix receptors can compensate for the loss of basal lamina components, suggesting that stronger adhesion to a less dense or structured basal ECM can still carry force across tissues [106].

On the other hand, directional feedback epidermal-muscle cells instructs the maintenance and the integrity of the three-dimensional epidermal structure and the overlying apical ECM. Accordingly, embryos defective for structural components of the epidermal CeHDs, like myotactin, VAB-10A, IFB-1, or MUP-4 all display impaired elongation from the two-fold stage onwards, similar to embryos defective for muscle contraction or adhesion to the basal ECM. Characteristically, these embryos show defects in the attachment of the epidermis to the underlying muscle cells, disrupting the path to force conduction [58,60,61,107,108,109]. In addition, the epidermis secretes its apical ECM, protecting the epidermal sheet from mechanical shearing stress by relaying force onto its cytoskeleton [69,71]. Consistent with this idea, the digestion of the embryonic sheath with trypsin results in embryos with disrupted form and halted elongation [75]. The same phenomenon is observed when individual components, such as LET-4, SYM-1, NOAH-1/2, or LPR-3, are absent from the composition of the apical ECM. These embryos display a characteristic halted elongation at the three-fold stage accompanied by rupture of the epidermis, indicative of an inability to cope with muscle produced forces (Figure 3D) [70,72,73]. Moreover, the discontinuity of the apical ECM in these embryos structurally destabilizes the CeHDs which results in the detachment of the musculature [71]. Despite lacking a physical connection, muscle function also impacts the embryonic sheath structure and remodeling. When muscle contractions (UNC-112) or sarcomere formation (PAT-3) are compromised, the continuity of the embryonic sheath is disrupted, and it often ruptures [71].

Most of these studies have focused on the direct link between musculature and the dorsal-ventral epidermis. Recently, however, the effect of muscles upon the seam cells is being revealed. Between the one-fold and two-fold stages, the par module concentrates at the CeAJs between the seam cells generating a planarly polarized tissue. This planar polarization is required for the dorsal-ventral orientation of the actin filaments and promotes the elongation of seam cells along the same axis. Surprisingly, and despite a lack of physical connection, the continuous path of force conduction from muscle cells into the dorsal-ventral epidermis is required for the maintenance of planar polarization at the seam cells [63]. When muscles cannot contract (UNC-112) or when their connection to the dorsal-ventral epidermis is disrupted (VAB-10A), the seam cells initially acquire planar polarization but it is not maintained. Consequently, the actin filaments do not align in a dorsal-ventral direction hampering their elongation along this axis [63]. The authors hypothesized that the mechanotransduction between the contacting epidermal tissues occurs at the level of the CeAJs, as loss of HMP-1 also results in defects in seam cell planar polarization [63]. Feedback reciprocity also seems to occur between seam cells and the muscles, contributing to the fine-tuning of muscle polarization. Loss of function of the myosin activation branch LET-502 and MLC-4, which mainly act at the level of the seam cells, generates rounder enlarged shaped muscles rather than spindly shaped ones, indicating a loss of proper polarization [100]. Altogether, the muscle cells and the epidermal tissue orchestrate the translation of forces into shape changes. When the embryo is vigorously rolling within the eggshell and reaches its three-fold in length, the exoskeleton of the animal, the cuticle, starts to be produced and secreted by the epidermis. The cuticle is a resistant, yet flexible layer constituted mainly by collagen. The cuticle performs many functions, including pathogen protection, osmolarity regulation or shaping the animal body [110]. In fact, cuticle defects have an impact early on in the life of the animal. The absence of collagen production or proteins required for the assembly of the cuticle structure such as SQT-3 impacts elongation. Despite reaching the three-fold stage, the hatching embryos retract to a non-elongated state, suggestive of a role for the cuticle in the rigidity of the final form of the larvae (Figure 3E) [75,111,112].

## 3. At Last

In summary, the epidermal tissue shapes the embryo into its final form through a combination of autonomous and non-autonomous mechanisms. The enclosure of the embryo relies on cell-autonomous actin dynamics and efficient adhesion, as well as the appropriate morphogenesis of the underlying neuroblast tissue. Likewise, embryonic elongation is dependent on cell autonomous actomyosin contractile forces but also on the forces generated by the underlying musculature. The understanding of how the epidermis shapes the initial oval embryo has come a long way, yet much remains unanswered: Do neuroblasts and muscle cells have epidermal morphogenetic functions beyond their ventral enclosure and elongation roles respectively? Which and in what way do other tissues help the epidermis to drive embryonic elongation? Does the eggshell provide substrate or signaling cues for epidermal morphogenesis? Do not miss the next episode, we surely will not!
